# Correlation between serum methotrexate-polyglutamate 3 (MTX-PG3) level and disease activity in rheumatoid arthritis patients: A prospective cohort study

**DOI:** 10.12688/f1000research.108714.1

**Published:** 2022-02-15

**Authors:** Eva Musdalita, Rudy Hidayat, Sumariyono Sumariyono, Suryo Anggoro Kusumo Wibowo, Anna Ariane, Hamzah Shatri, Iris Rengganis, Dono Antono

**Affiliations:** 1 Division of Rheumatology, Department of Internal Medicine, Faculty of Medicine,, Universitas Indonesia/ Dr Cipto Mangunkusumo General Hospital, Jakarta, Indonesia; 2 Department of Internal Medicine, Faculty of Medicine, Universitas Syiah Kuala, Banda Aceh, Indonesia; 3 Division of Psychosomatic and Palliative Care, Department of Internal Medicine, Faculty of Medicine, Universitas Indonesia/Dr Cipto Mangunkusumo General Hospital, Jakarta, Indonesia; 4 Division of Allergy and Immunology, Department of Internal Medicine, Faculty of Medicine, Universitas Indonesia/Dr Cipto Mangunkusumo General Hospital, Jakarta, Indonesia; 5 Division of Cardiovascular, Department of Internal Medicine, Faculty of Medicine, Universitas Indonesia/Dr Cipto Mangunkusumo General Hospital, Jakarta, Indonesia

**Keywords:** Rheumatoid arthritis, MTX-PG3 level, disease activity, DAS28-CRP, methotrexate

## Abstract

**Background:** Rheumatoid arthritis (RA) is one of the most common autoimmune diseases, characterized by systemic inflammation, joint destruction and disability. Methotrexate (MTX) is used as the primary treatment for RA patients. However, the response to MTX therapy is highly varied and difficult to predict. This study sought to determine the role of MTX by measuring the MTX polyglutamate 3 (MTX-PG3) levels and the disease activity score 28 based on C-reactive protein (DAS28-CRP) of RA patients.

**Method:** A prospective cohort study was conducted at the Rheumatology Polyclinic of Dr. Cipto Mangunkusumo General Hospital. Thirty-four patients with RA were included and followed up to 12 weeks. The RA patients were treated with MTX 10 mg per week and an increased dose of 5 mg per week every month. DAS28-CRP and MTX-PG3 level were assessed at week 8 and 12. Multivariate logistic regression analysis was used to determine the correlation between MTX-PG3 and DAS28-CRP.

**Result:** A total of 34 RA patients were followed and the MTX was well tolerated in which no increase of serum glutamic oxaloacetic transaminase (SGOT), serum glutamic pyruvic transaminase (SGPT) and glomerular filtration rate (GFR) were observed. The mean scores of DAS28-CRP decreased following the MTX-treatment: 3.93, 3.22 and 2.82 at week 0, 8 and 12, respectively. In contrast, the median concentration of MTX-PG3 increased from week 8 to week 12 followed by increasing the dose of MTX. Our analysis suggested there was a moderate positive correlation between MTX-PG3 levels and DAS28-CRP score at week 8 and week 12 post-MTX treatment.

**Conclusion: **The level of MTX-PG3 is correlated with DAS28-CRP score suggesting that MTX-PG3 could be used as an indicator to assess the disease activity in RA patients. Nevertheless, a prospective study with a higher number of patients is needed to confirm this finding.

## Introduction

Methotrexate (MTX) is the first-line drug to treat rheumatoid arthritis (RA) which provides higher survival rate than other disease modifying arthritis rheumatoid drugs (DMARD) and is recommended by the European League Against Rheumatism and the American College of Rheumatology. MTX has become a commonly used treatment option because it is a cost-effective and has acceptable safety profile. ^1^^–^^3^ Although MTX is the main RA therapy and most patients show symptomatic improvement and have acceptable side effects, the response to MTX therapy is highly varied and difficult to predict. ^4^

MTX is transported into cells via the reduced folate carrier pathway and activated by polyglutamate synthase to methotrexate polyglutamate (MTX-PG). ^5^ MTX-PG then inhibits the target enzymes such as thymidylate synthetase *,* dihydrofolate reductase and key enzymes for *de novo* purine synthesis pathway. ^6^ Depending on the number of conjugated glutamates, MTX-PG may be present as MTX-PG1-5 or the longer chain MTX-PG (MTX-PG3-5) which is considered to be more active. MTX-PG3 is the most dominant and stable type of MTX-PG and could reflect the overall polyglutamate status. ^7^ Therapeutic effects of MTX on RA patients depends on conversion of MTX to MTX-PG and therefore intracellular measurement of MTX-PG has been proposed as an objective method of guiding MTX therapy. ^8^

Studies have been conducted to analyze the pharmacodynamics of MTX-PG, in particular MTX-PG3, since it is expected to be used to monitor the safety and effectiveness of MTX therapy in RA patients. ^8^^,^^9^ A prospective cohort study found that MTX-PG3 and total MTX-PG levels were associated with decreased disease activity score 28 (DAS28) as measured at 3, 6, and 12 weeks. MTX-PG3 levels also positively correlated with the number of MTX dose during the treatment. ^9^ However, studies showed different results in which the levels of MTX-PG3 had no potential as a marker of clinical improvement in RA patients, and also there was no significant relationship between MTX-PG level with the side effects and treatment response status. ^10^^,^^11^ Therefore, the MTX-PG concentration in erythrocytes as a predictor of response to MTX therapy in RA patients is still a conflict. Besides that, the cut-off value for MTX-PG levels is different in each country since RA is determined by specific genetic factors. ^12^ This prospective study aimed to clarify the relationship between intra erythrocytic MTX-PG3 concentration and DAS28 based on C-reactive protein (DAS28-CRP) in Indonesia. The CRP value is considered more sensitive than short-term changes in RA disease activity. ^13^ This study was expected to provide a better understanding of the role of MTX-PG3 in the treatment of RA.

## Methods

### Study design and patients

A prospective cohort study was conducted among RA patients treated at Rheumatology Polyclinic of Dr. Cipto Mangunkusumo General Hospital (RSCM) in Jakarta. The RA patient was diagnosed based on the 2010 American College of Rheumatology/European League Against Rheumatism (2010 ACR/EULAR) diagnostic criteria which is a new and more sensitive approach in diagnosing RA. The diagnostic criteria of 2010 ACR/EULAR includes joint involvement, serological test, CRP, erythrocyte sedimentation rate (ESR), and duration of symptoms. ^14^ Before MTX treatment, DAS28-CRP was assessed, demographic and clinical data were collected including gender, age, body mass index (BMI), CRP, nonsteroidal anti-inflammatory drugs (NSAID) use, steroid use, serum glutamic oxaloacetic transaminase (SGOT), serum glutamic pyruvic transaminase (SGPT), glomerular filtration rate (GFR) and erythrocyte sedimentation rate (ESR). Subsequently, the patients were treated with MTX and were followed until 12 weeks post-treatment. The DAS28-CRP examination and the concentration of MTX-PG3 were re-assessed at week 8 and 12.

### MTX treatment and follow-up

The patients received MTX therapy with an initial dose of 10 mg/week and an increased dose of 5 mg/week every month according to the degree of disease activity. The patients also received methylprednisolone 4 mg twice daily and omeprazole 20 mg once daily. At week 8 and 12, the venous blood was collected at least 1.5 hours after MTX administrated.

### DAS28-CRP assessment

The DAS28-CRP assessment was conducted before the MTX treatment and during the follow-up of 8 and 12 weeks post-treatment. DAS28-CRP assesses 28 tender and swollen joint counts, general health and the level of CRP (mg/L) and the score was calculated based on formula that have been provided elsewhere. ^15^ The DAS28-CRP score interpretation are as follows: <2.6: disease remission, 2.6–3.2: low disease activity, <3.2–5.1: moderate disease activity, and >5.1: high disease activity. ^16^

### MTX-PG3 measurement

To measure the MTX-PG3, the blood samples were first centrifuged for 5 min at 3000 rpm. ^17^ The level of MTX-PG3 was measured using chromatographic analysis by injecting 10 μL of sample into Waters ^TM^ ACQUITY UPLC BEH C18 column (Waters Corporation, Milford, MA, USA) with mobile phase of 10 mM ammonium bicarbonate at pH 10, ammonium hydroxide, and methanol. The rate was maintained at 0.3 mL/min and analysis was conducted using the program of 0-0.5 min isocratic hold 5% B, 0.5-4 min linear gradient 5-40% B; 4.0-4.25 min linear gradient 4-100% B; 4.25-4.75 min isocratic 95% B; 4.75-5 min linear gradient 100-5% B; and 5-6 min 5% isocratic.

### Statistical analysis

Descriptive analysis was performed to provide the distribution of the data. The MTX-PG3 levels and DAS28-CRP score were presented as mean ± standard deviation if the data normally distributed otherwise as median (min-max). At univariate analysis, Spearman’s correlation test was used to assess the correlation between the potential confounding variables with DAS28-CRP. The multivariate linear regression test was used to determine the correlation between the MTX-PG3 levels and DAS28-CRP scores by controlling for confounding variables. All analyses were conducted using SPSS software version 24 (SPSS Inc., Chicago, IL, USA, RRID:SCR_002865).

## Results

### Patients’ characteristics

A total of 34 RA patients were enrolled and analyzed, and the characteristics of the patients are presented in Table 1. No patients dropped out from the follow-up. Most of the patients (94.1%, 32/34) were female with an age range of 20 to 70 years. The mean BMI was 23.29 kg/m ^2^, indicating that most of the patients were in the ideal weight range. All patients did not smoke and had no comorbidities. Examination of SGOT, SGPT, and GFR showed normal limits with medians of 18 U/L, 18 U/L, and 102.5 mL/min/1.73m ^2^, respectively. The median of disease duration was 8.5 months.

**Table 1.** Demographic and clinical characteristics (n=34).

Characteristics	Mean (SD)
Age (year), median (min-max)	48 (20–70)
Gender (n, %)	
Male	2 (5.9)
Female	32 (94.1)
Body mass index (kg/m ^2^)	23.26 (0.73)
Current smoker (n, %)	0 (0)
Comorbid, (n, %)	0 (0)
GFR (mL/min/1.73m ^2^)	102.5 (3.5)
SGOT (U/L), median (min-max)	18 (10–120)
SGPT (U/L), median (min-max)	18 (9–61)
CRP (mg/L), median (min-max)	3.0 (0.1–28.01)
Number of joints, median (min-max)	9 (9–23)
Disease duration (month), median (min-max)	8.5 (7–13)
DAS28-CRP	
Week 0	3.93 (0.137)
Week 8	3.22 (0.148)
Week 12	2.82 (0.165)
ΔDAS28-CRP	
Week 0-8	0.54 (0.063)
Week 8-12	1.03 (0.071)
MTX-PG3 (nmol/L)	
Week 8 Median (min-max)	12.63 (1.33–81.30)
Week 12 Median (min-max)	42.67 (4.59–156.35)

The mean score of DAS28-CRP before the MTX treatment was 3.93 indicating most of the patients had a moderate disease activity state, then the DAS28-CRP scores decreased to 3.22 and 2.82 at week 8 and 12, respectively. The data of MTX-PG3 levels were not normally distributed with median at week 8 and 12 being 12.63 nmol/L and 42.67 nmol/L, respectively.

### Correlation between MTX-PG3 and DAS28-CRP

A linear regression was used to identify the correlation of MTX-PG3 with DAS28-CRP and the changes of DAS28-CRP score (ΔDAS28-CRP). To do this, the confounding variables were first assessed including BMI, age, GFR, MTX dose, duration of disease, methylprednisolone dose, and number of joints affected through the Spearman’s correlation test. This analysis suggested that disease duration, methylprednisolone dose, and number of joints were potential confounding variables and therefore adjusted for further analysis ( Table 2).

**Table 2.** Spearman’s correlation showing the potential confounding variables associated with DAS28-CRP.

Variable	DAS28-CRP at week 8		DAS28-CRP at week 12	
	r	p-value	r	p-value
Disease duration	0.299	0.930	0.218	0.231
Methylprednisolone dose at week 8	0.564	0.001	0.336	0.060
Methylprednisolone dose at week 12	0.287	0.111	0.456	0.007
The number of joints involved	0.351	0.042	0.307	0.087

r: Spearman’s correlation coefficient.

The multivariate analysis of the role of MTX-PG3 on DAS28-CRP with a linear regression are provided in Table 3. There was a significant correlation between MTX-PG3 levels and DAS28-CRP score at week 8 and 12 post-MTX treatment after adjusted all confounding variables, r=0.517 with p=0.022 and r=0.538 with p=0.013, respectively. Our analyses also suggested that there were significant correlations between the level of MTX-PG3 and the changes of DAS28-CRP scores (ΔDAS28-CRP) at week 8 and 12 post-MTX treatment (r=0.933 and r=0.787, respectively with p<0.001 for both) ( Table 3).

**Table 3.** Multivariate analysis showing the correlation of MTX-PG3 level with DAS28-CRP and ΔDAS28-CRP.

Variable	Crude R ^2^	Adjusted R ^2^	p-value
DAS28-CRP			
MTX-PG3 at week 8	0.719	0.517	0.022
MTX-PG3 at week 12	0.734	0.538	0.013
ΔDAS28-CRP			
MTX-PG3 at week 8	0.966	0.933	<0.001
MTX-PG3 at week 12	0.935	0.787	<0.001

R ^2^: Coefficient of determination.

### Receiver Operating Characteristics (ROC) analysis

**Figure 1.**
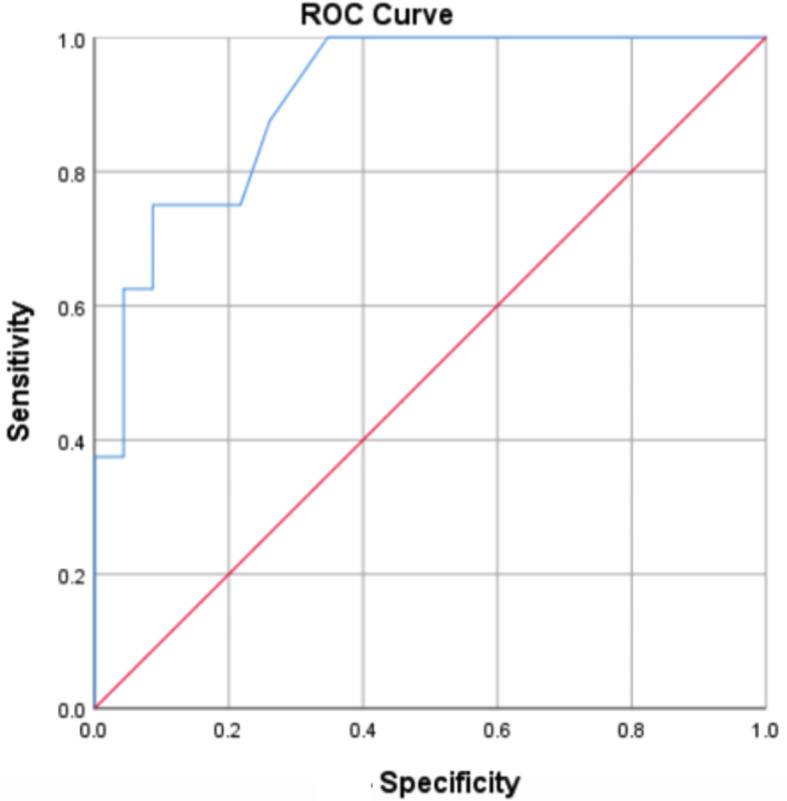
The sensitive and specific of the MTX-PG3 level to determine the change of DAS28-CRP score.

ROC analysis was conducted using ROC curve to determine how sensitive and specific the MTX-PG3 level was to predict the disease activity of RA. Data from 12 weeks post-treatment suggested that the cut-off value of MTX-PG3 level 78.4 nmol/L was able to reduce the DAS28-CRP score by more than 1.2 times. The area under the curve (AUC) was 0.9 (95% confidence interval 0.81–1, p=0.001) with a sensitivity of 75% and a specificity of 91% ( Figure 1).

## Discussion

In this study, we treated the RA patients with 10 mg MTX as an initial dose followed by an increase of 5 mg MTX/week, every month. A previous study found that treatment with an initial dose of at least 10 mg/week for the first three months with an increase of at least 20 mg/week for six months gave an excellent clinical response. ^18^ Our data suggested that by increasing the MTX dose the MTX-PG3 level could increase by 70.4% from week 8 to week 12. In contrast, the DAS28-CRP score decreased from 3.93 (first week) to 3.93 at week 12, indicating that the disease activity changed from moderate to low degree. Based on the changes of DAS28-CRP scores, the response of the treatment was categorized as moderate response. ^17^^,^^19^ In this study, MTX dose and MTX-PG3 levels were well tolerated since no adverse events presented at week 8 or 12, as indicated by no increase in the levels of SGOT, SGPT and GFR reported.

Our data suggested that the levels of MTX-PG3 were correlated with DAS28-CRP score at week 8 and 12 post-MTX treatment. The same results were also observed between MTX-PG3 and ΔDAS28-CRP. These suggested that the higher MTX-PG3 levels the higher the score of DAS28-CRP (i.e., the better the disease activity). These results are similar to a study in Japanese patients in which there was a significant association between MTX-PG3 level and DAS28 score from week 8 to 24 post-MTX therapy. ^17^ The decrease in DAS28-CRP may occur in the folate reduction pathway through dihydrofolate reductase (DHFR) inhibition. Reduced DHFR activity interferes with homocysteine conversion to methionine and causes inhibition of immunoglobulin and rheumatoid factor inducers. ^20^^–^^22^

Further analysis using the ROC curve showed that MTX-PG3 level of 78.4 nmol/L at 12 weeks post-MTX treatment could predict a good response of the MTX treatment. A previous study reported that the cut-off level of MTX-PG at week 12 was more than 74 nmol/L to reduce DAS28 score and categorized as a moderate/good response. ^9^

There are some limitations of this study. The number of the patients included in this study were relatively small and a further study with a larger sample size and longer follow-up time is required. It should be noted that the correlation of the MTX-PG3 level with DAS28-CRP score and ΔDAS28-CRP was influenced by confounding variables including duration of disease, methylprednisolone dose, and number of joints involved. Confounding factors are the main concern in the observational cohort study since no randomization was conducted. ^23^ Nevertheless, we have tried to minimize the bias by adjusting those co-founding in the final analysis. In addition, several studies have shown that genetic factors play a role in the variability of treatment of each patient, in particular variations on gene encoding enzymes in the folate metabolism pathway. ^5^^,^^24^ In this study, genetic factors were not controlled and could be the source of bias.

## Conclusion

Our study suggests a moderate positive correlation between MTX-PG3 level and DAS28-CRP score in RA patients after 8 and 12 weeks post-MTX treatment. This indicates that level of MTX-PG3 may potentially be used to predict the degree of RA disease activity and as an indicator of the patient response to MTX treatment.

## Data availability

### Underlying data

Figshare: Correlation between serum methotrexate-polyglutamate 3 (MTX-PG3) level and disease activity in rheumatoid arthritis patients: A prospective cohort study https://doi.org/10.6084/m9.figshare.18079136.

Data are available under the terms of the Creative Commons Attribution 4.0 International license (CC-BY 4.0).

### Reporting guidelines

Figshare: STROBE checklist for: ‘Correlation between serum methotrexate-polyglutamate 3 (MTX-PG3) level and disease activity in rheumatoid arthritis patients: A prospective cohort study’, https://doi.org/10.6084/m9.figshare.18743006.

Data are available under the terms of the Creative Commons Attribution 4.0 International license (CC-BY 4.0).

## Ethics statement

The protocol of the study was approved by the Ethics Committee of the Faculty of Medicine, Universitas Indonesia and Dr Cipto Mangunkusumo General Hospital (KET-1144/UN2.F1/ETIK/PPM.00.02/2020). All patients provided written informed consent prior to participate in this study. All works were conducted in accordance with The Code of the World Medical Association (Declaration of Helsinki).
