# Supplementary material for: Correlation between serum methotrexate-polyglutamate 3 (MTX-PG3) level and disease activity in rheumatoid arthritis patients: A prospective cohort study
Source: F1000Res. 2022 Feb 15;11:187. [Version 1] doi: 10.12688/f1000research.108714.1 (PMC8891717; doi:10.12688/f1000research.108714.1)
Supplement: Peer review history

# Peer review: Reviewer response for version 1

**DOI:** 10.5256/f1000research.120131.r123624

**Published:** 2022-03-02

**Authors:** Talha Bin Emran

**Affiliations:** 1 Department of Pharmacy, BGC Trust University Bangladesh, Chittagong, Bangladesh

Title: Correlation between serum methotrexate-polyglutamate 3 (MTX-PG3) level and disease activity in rheumatoid arthritis patients: A prospective cohort study

**Minor comments:**

 Although the article has scientific rigor, several minor flows need to be improved before publication:

 1. The abstract section is unsuitable—no focus point in the abstract section.

 2. "Nevertheless, a prospective study with a higher number of patients is needed to confirm this finding." Is this necessary?

 3. Authors are suggested to use the full form when used for the first time throughout the manuscript.

 4. The aim of the study should be written as the last paragraph of the introduction.

 7. MTX treatment and follow-up: How was this selected?

 8. Receiver Operating Characteristics (ROC) analysis: Please describe in further detail.

 9. "Further analysis using the ROC curve showed that MTX-PG3 level…" needs more insights with relevant references.

 10. Presentation of figures is good.

 11. Figure legends are appropriate and self-explanatory.

 12. The conclusion needs to address future perspectives.

 13. Spacing, punctuation marks, grammar, and spelling errors should be reviewed thoroughly.

Is the work clearly and accurately presented and does it cite the current literature?

Yes

If applicable, is the statistical analysis and its interpretation appropriate?

Yes

Are all the source data underlying the results available to ensure full reproducibility?

Yes

Is the study design appropriate and is the work technically sound?

Yes

Are the conclusions drawn adequately supported by the results?

Yes

Are sufficient details of methods and analysis provided to allow replication by others?

Yes

Reviewer Expertise:

Public health, Immunology, Natural Product Chemistry

I confirm that I have read this submission and believe that I have an appropriate level of expertise to confirm that it is of an acceptable scientific standard, however I have significant reservations, as outlined above.

# Peer review: Reviewer response for version 1

**DOI:** 10.5256/f1000research.120131.r123627

**Published:** 2022-02-23

**Authors:** Andri Frediansyah

**Affiliations:** 1 Research Division for Natural Product Technology, Indonesian Institute of Sciences (LIPI), Wonosari, Indonesia

The researchers looked at 34 people with rheumatoid arthritis (RA) to see if there was a link between MTX-PG levels and how active their RA was. There were two women and 32 men in the study. The subject matter is of general interest, and the study yields useful information. There are, however, a few issues that should be addressed:

 1) Please specify the date, duration, and months of the experiment.

 2) Please verify the following statement: "low disease activity, <3.2–5.1". Is this correct?

 3)The methods section is unclear. Please describe it in detail. Is there a particular type of blood (whole blood, red, or white blood cells) that you used in the study? Additionally, please provide detailed information about the centrifugation parameters, such as time, temperature, and g-force/RCF (g). Prior to analysis, is the blood subjected to any special treatment?

 4) Please rewrite the section on chromatography measurement and analysis in detail. Include the HPLC specification and brand; column details (including particle size, pore size, inner diameter, and length); ammonium hydrochloride concentration and pH; solvent B composition (or A, if any); and the reference you cited.

 5) Did you combine ammonium bicarbonate and ammonium chloride, and if so, in what proportion? Which detector (UV/CAD/MS) did you use? If UV/DAD, at what wavelength did you adjust the detector?

 6) Please specify the brand of the MTX-PG3 standard and the R2 (nmol) value of the standard you used.

Is the work clearly and accurately presented and does it cite the current literature?

Yes

If applicable, is the statistical analysis and its interpretation appropriate?

I cannot comment. A qualified statistician is required.

Are all the source data underlying the results available to ensure full reproducibility?

Yes

Is the study design appropriate and is the work technically sound?

Yes

Are the conclusions drawn adequately supported by the results?

Yes

Are sufficient details of methods and analysis provided to allow replication by others?

Partly

Reviewer Expertise:

Pharmaco Biology/Analytical Chemistry

I confirm that I have read this submission and believe that I have an appropriate level of expertise to confirm that it is of an acceptable scientific standard, however I have significant reservations, as outlined above.

# Peer review: Reviewer response for version 1

**DOI:** 10.5256/f1000research.120131.r123625

**Published:** 2022-02-21

**Authors:** Slobodan M. Janković

**Affiliations:** 1 Faculty of Medical Sciences, Department of Pharmacy, University of Kragujevac, Kragujevac, Serbia

The authors made an observational study trying to correlate MTX PG levels with disease activity of RA (as measured by a clinical score). The topic is of general interest, and the study brings results with practical significance. The manuscript is well written, and merits acceptance for publication. However, there are a few issues that should be corrected:

1. In the Methods section the authors should state precisely how they measures the MTX PG levels in erythrocytes. As it is written now, it is not clear whether the MTX PG levels were measured in erythrocytes or in full blood.
2. Number of patients is small, so it is critical that statistical methods were used properly. The authors should state whether assumptions of multivariate logistic regression were met. Also, what was the categorical outcome used as dependent variable of the regression? Finally, quality of the regression model should be stated (Hosmer Lemeshow test, Cox and Snellen...).
3. Something should be said about adherence of the patients to the therapy. Was there any method used to check for adherence? If not, mention this in the Limitation paragraph.

Is the work clearly and accurately presented and does it cite the current literature?

Yes

If applicable, is the statistical analysis and its interpretation appropriate?

Partly

Are all the source data underlying the results available to ensure full reproducibility?

Yes

Is the study design appropriate and is the work technically sound?

Yes

Are the conclusions drawn adequately supported by the results?

Yes

Are sufficient details of methods and analysis provided to allow replication by others?

Yes

Reviewer Expertise:

Clinical pharmacology

I confirm that I have read this submission and believe that I have an appropriate level of expertise to confirm that it is of an acceptable scientific standard.
